# Mechanical characteristics of bacterial cellulose-reinforced mycelium composite materials

**DOI:** 10.1186/s40694-021-00125-4

**Published:** 2021-12-04

**Authors:** Elise Elsacker, Simon Vandelook, Bastien Damsin, Aurélie Van Wylick, Eveline Peeters, Lars De Laet

**Affiliations:** 1 Architectural Engineering Research Group, Department of Architectural Engineering, Vrije Universiteit Brussel, Pleinlaan 2, 1050 Brussels, Belgium; 2 Research Group of Microbiology, Department of Bioengineering Sciences, Vrije Universiteit Brussel, Pleinlaan 2, 1050 Brussels, Belgium; 3 Present Address: Newcastle University, Hub for Biotechnology in the Built Environment, Devonshire Building, Newcastle upon Tyne, NE1 7RU UK

**Keywords:** Mycelium materials, *Trametes versicolor*, White-rot fungi, Bacterial cellulose, Biocomposite, Hybrid materials

## Abstract

**Background:**

While mycelium is considered a promising alternative for fossil-based resins in lignocellulosic materials, the mechanical properties of mycelium composite materials remain suboptimal, among other reasons due to the weak internal bonds between the hyphae and the natural fibres. A solution could be provided by the hybridisation of mycelium materials with organic additives. More specifically, bacterial cellulose seems to be a promising additive that could result in reinforcing mycelium composites; however, this strategy is underreported in scientific literature.

**Results:**

In this study, we set out to investigate the mechanical properties of mycelium composites, produced with the white-rot fungus *Trametes versicolor*, and supplemented with bacterial cellulose as an organic additive. A methodological framework is developed for the facile production of bacterial cellulose and subsequent fabrication of mycelium composite particle boards based on a hybrid substrate consisting of bacterial cellulose and hemp in combination with a heat-pressing approach. We found that, upon adding bacterial cellulose, the internal bond of the composite particle boards significantly improved.

**Conclusions:**

The addition of bacterial cellulose to mycelium composite materials not only results in a strengthening of internal bonding of mycelium material, but also renders tuneable mechanical properties to the material. As such, this study contributes to the ongoing development of fully biological hybrid materials with performant mechanical characteristics.

## Background

Research  about the development of renewable, biodegradable and eco-friendly materials has seen a growing interest as alternatives to synthetic materials are key to reduce anthropogenic impact on suffering ecosystems. Lignocellulosic fibres are an appealing feedstock for such bio-based substitutes as they allow for the valorisation of existing agricultural side waste streams. While lignocellulose-based materials are classically made by using formaldehyde-based resin binders, which are fossil-derived, toxic and require energy-intensive conditions to be produced [1], recent focus has shifted towards replacing this with biological binders that are more sustainable, such as mycelium [2, 3]. An additional advantage is that mycelium is in situ produced by means of biological growth. Soil-associated filamentous fungi have the natural capability to degrade (ligno-)cellulosic biomass [4]. The three-dimensional interwoven hyphal network that is formed serves as a natural glue and binds the feedstock to form a unified and lightweight composite material [2]. After substrate colonisation, this composite is heated to kill the fungal organism and remove the moisture [5]. Several basidiomycetes species with a saprotrophic lifestyle have been shown to yield mycelium-based materials, with *Trametes versicolor*, *Ganoderma lucidum*, *Pleurotus ostreatus* and *Schizopyllum commune* being the most commonly used [2].

Despite the promise of mycelium-based composites, applications remain limited because of suboptimal mechanical properties [2, 6, 7]. More specifically, the tensile strength is typically too low and should be subject to improvement. This can be explained by the agricultural residue fibres often having a low strength due to their processing [5] and by the mycelium network being characterized by an intrinsic weak bonding with the fibres at the molecular level. Therefore, additional binders are added, aimed to improve the mechanical properties of the material. Cellulose nanofibril binders, which are isolated from the most abundant organic compound on Earth, cellulose [8, 9], showed promising properties [10], such as high mechanical strength [11–14]. Considerable attention is given to cellulose nanofibril, because these have a high surface area and can bond natural fibres through hydrogen bonding and mechanical interlocking, providing structural integrity to the composites [15–21]. Indeed, the incorporation of cellulose nanofibrils into natural polymers has proven to be an essential strategy for developing bio-based materials [10, 13, 22]. Nanocellulose can be produced from various lignocellulosic sources through different methods. The classical production method, consisting of delamination of wood pulp by supplying mechanical shear in combination with chemical and/or enzymatic treatments [23]. Depending on the type of processing and raw material, the method is quite energy-intensive and requires chemicals for fibrillation pre-treatment [11, 24].

A potential alternative that requires fewer processing steps is bacterial cellulose (BC), which is naturally synthesised by certain Gram-negative bacteria. BC constitutes a promising biomaterial due to several advantages: it is easy to obtain in a highly pure state, is biodegradable and is characterized by a high stiffness and tensile strength [25, 26], low density [27] and an easily manipulable shape [28]. BC’s structural features are even superior to those of plant cellulose [29, 30], including higher water holding capacity, higher crystallinity, greater tensile strength, an ultrafine fiber network and the ability to be molded into various shapes during production. A well-described BC-producing species is *Komagataeibacter xylinus* [31], which has an aerobic chemoorganotrophic fermentative metabolism and uses various carbon and nitrogen sources [32]. Besides *Komagataeibacter spp.*, the *Acetobacteraceae* family consists of multiple genera that are capable of producing BC [33]. The biological function of BC production is the creation of a biofilm during fruit colonisation, thereby protecting cells from desiccation and UV damage. In the laboratory, bacteria are grown in liquid culture in static conditions and reside at the air–water interface, resulting in the production of a pellicle of intertwined cellulose fibrils [34]. BC materials have been implemented in many applications, including binding agents [35], cosmetics [36], high-quality paper [35], food [36], textiles [34], tissue engineering scaffolds [37, 38] and nanocomposites [28, 35, 39]. The bacteria can also grow on the surface of natural fibres rather than in a liquid medium [40]. The adhesion between BC nanofibrils and natural fibres can possibly be attributed to hydrogen bond formation between the BC and the natural fibres [35].

In this work, we set out to generate more insights into the hybridisation of mycelium composites with BC by developing fully bio-fabricated and biodegradable composite materials made from natural fibres such as hemp, BC and mycelium. As defined by Drisko and Sanchez et al., with hybridization, two dissimilar components are blended to make a single entity with either enhanced or completely new properties [41]. We aim to demonstrate the feasibility of an *in-situ* fabrication approach, while establishing a methodology to explore a sequence of enhancements of mycelium materials. We investigate the hypothesis that the addition of BC might lead to mechanically enhanced mycelium composites, as it is known that both the type of additive and post-treatments can influence the material properties of mycelium materials. Particle boards are manufactured and mechanically tested to determine their bending, tensile and internal bonding behaviour. The particle boards are manufactured from hemp chips, BC and mycelium as a binder. As such living BC is mixed with hemp fibres, after which mycelium is added to the substrate. The grown substrate is then compacted with a heat press. To our knowledge, this is the first study undertaking a mechanical analysis of BC-mycelium hybrid materials.

## Results

### Fabrication of BC-mycelium composite particle boards

**Fig. 1:**
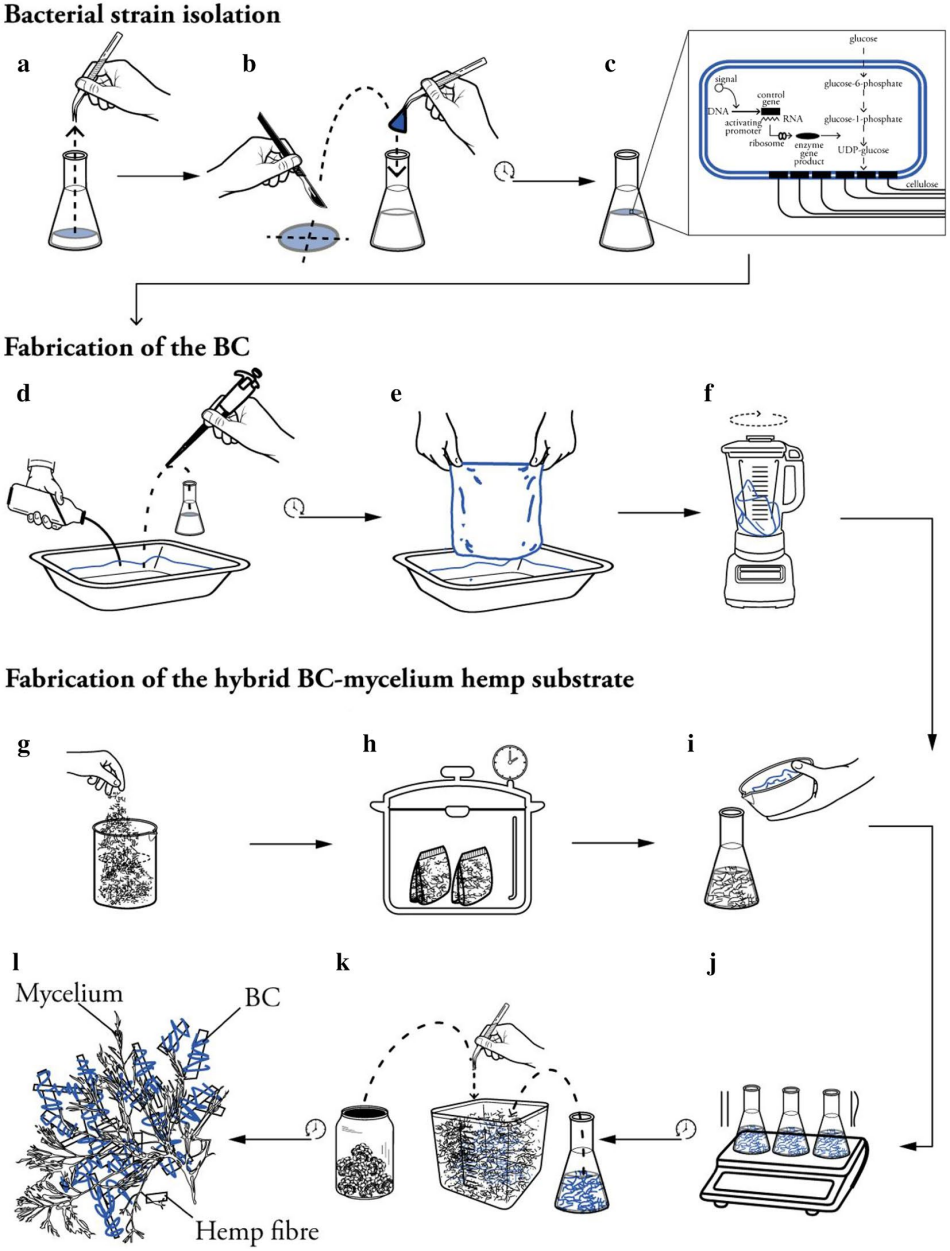
Schematic depiction of the experimental procedure of the fabrication of BC-mycelium composite material. **a** Isolation of a *K. xylinus* bacterial strain from a SCOBY. **b** Transfer of *K. xylinus* bacterial strain to HS culture medium. **c** Incubation of a *K. xylinus* preculture, during which BC is produced through a metabolic process. **d** Re-inoculation of a *K. xylinus* culture in a set-up enabling the production of larger BC sheets. **e** Rinsing of the obtained BC sheet with deionised water. **f** Mechanical disintegration of the BC sheet with a lab blender. **g** Preparation of dry hemp fibres in autoclave bags. **h** Sterilization of the he substrate. **i** Mixing of BC with hemp fibres. **j** Inoculation of the mixture. **k** Mixing of mycelium spawn with the BC-hemp substrate. **l** Formation of BC-mycelium composite material through hyphal growth

**Fig. 2:**
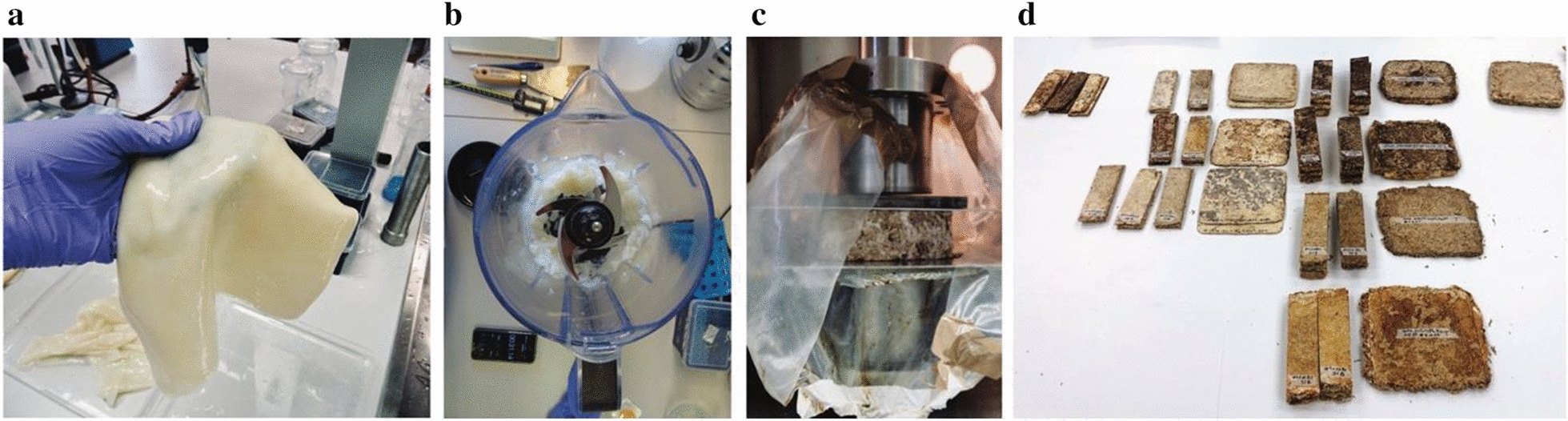
Crucial steps in the preparation of hybrid BC-mycelium composites. **a** An obtained BC sheet just after rinsing it with deionized water **b** Mechanically disintegrated BC pellicles in a lab blender. **c** Compression of BC-mycelium samples with an Instron having an oven built around. **d** Resulting particle boards cut to the specimen dimensions required for the different mechanical tests

To initiate this study, different BC-mycelium composite samples were prepared by harvesting pure BC sheets from *K. xylinus* cultures, mechanically disintegrating this BC and combing it with hemp to obtain a hybrid BC-hemp substrate. This substrate, in which the BC nanofibrils have presumably self-assembled repetitive building blocks into higher-order structures to form a network-like tissue around the hemp fibres, was then used to sustain growth of *T. versicolor* in a classical set-up for production of composite mycelium materials (Figs. 1 and 2a and b). In parallel, a mycelium composite lacking BC was prepared as a negative control. In all cases, *T. versicolor* mycelium homogenously colonised the substrate generating material samples shaped as the rectangular moulds, without any visible differences between pure mycelium and hybrid BC-mycelium samples. These samples were then converted to particle board samples by heat-press compression, either at 70 °C or at 200 °C (only at 70 °C for the negative control) and cut into smaller specimens ready for mechanical characterization (Fig. 2c and d).

### Bending behaviour of BC-mycelium composites

**Fig. 3:**
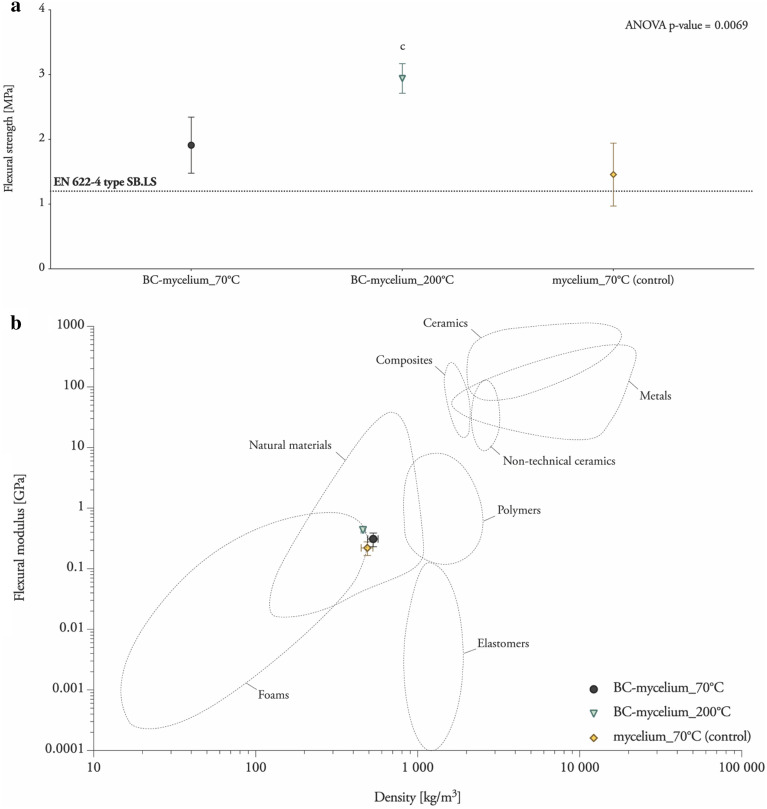
Bending behaviour of BC-mycelium composite particle boards. **a** Flexural strength of BC-mycelium composites. The horizontal line indicates the minimum requirement for the bending strength to meet the European standard EN 622-4 for load-bearing use for soft boards (type SB.LS). **b** Flexural modulus–density chart of BC-mycelium composites plotted on the Ashby chart for engineering materials [56]

As a first step towards assessing the mechanical performance of BC-mycelium composite particle boards, all particle board samples were subjected to bending behaviour analysis using a three-point static flexural test (Table 1; Fig. 3). As compared to a control sample lacking BC, an improved mean flexural strength and modulus was observed for samples containing BC. Yet, there is no significant differences in the flexural strength between *BC-mycelium_70°C* and *mycelium_70°C*. Increasing the heat to 200 °C during the densification process even improved the flexural strength of the samples (*BC-mycelium_200°C*) by 200% compared with the control sample (*mycelium_70°C*), and by 150% compared with samples densified at 70 °C (*BC-mycelium_70°C*) (Table 1; Fig. 3). Moreover, based on these characteristics, BC-mycelium materials exceed the minimal requirements for soft boards as defined in EN 622-4 (2019) (type SB.LS) in dry, humid and exterior conditions and load-bearing use (Fig. 3a). It can be concluded that the flexural properties of BC-mycelium composite materials are similar to those of natural materials such as cork and wood (Fig. 2b).

**Table 1:** Overview of the material properties revealed by three-point bending tests of BC-mycelium composites

Label	Dry density [kg/m^3^]	Flexural strength [MPa]	Flexural modulus [GPa]
*BC-mycelium_70°C*	531.17 ± 29.08	1.91 ± 0.43	0.31 ± 0.08
*BC-mycelium_200°C*	460.30 ± 12.66	2.94 ± 0.23	0.44 ± 0.02
*mycelium_70°C* (control)	488.89 ± 41.09	1.46 ± 0.48	0.22 ± 0.06

The standard deviation was based on measurements of triplicate specimens (mean ± one standard deviation)

### Tensile properties of pure BC sheets and BC-mycelium composites parallel to the surface

**Fig. 4:**
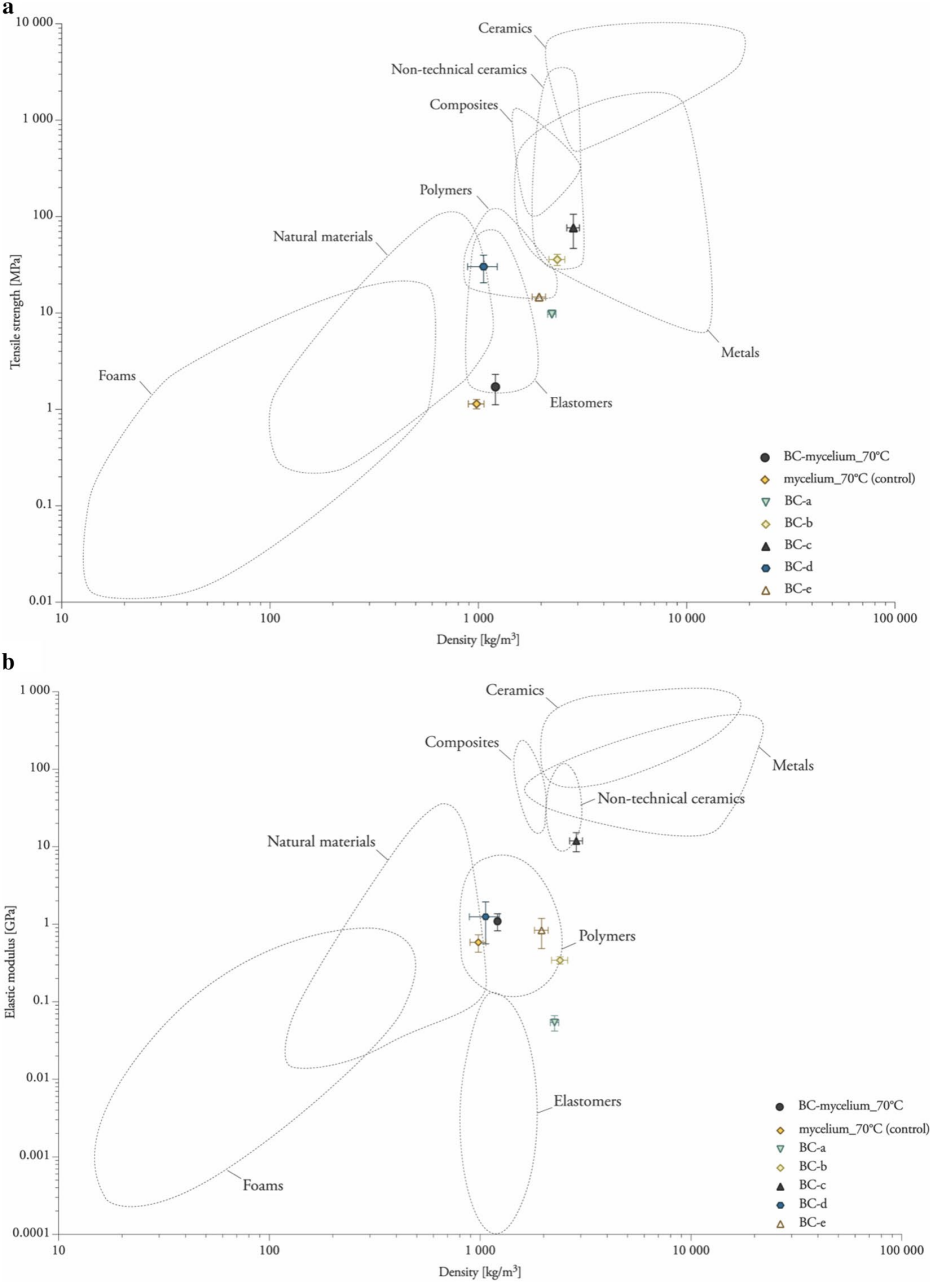
Tensile properties parallel to the surface. **a** Tensile strength–density chart of BC-mycelium composites. **b** Elastic modulus–density chart of bacterial cellulose mycelium composite, plotted on the Ashby chart for engineering materials [56]

In a next phase of the research, we set out to investigate the tensile properties of the materials (Table 2; Fig. 4). As a reference, pure BC samples were also subjected to this test and, with the aim of analysing the influence of the drying process on the tensile properties, five different treatments were performed with these samples ranging from no treatment at all to heat-pressing and blending.

**Table 2:** Overview of the material properties in the tension of BC-mycelium composite materials and pure BC materials

Label	Dry density [kg/m^3^]	Ultimate strength σ [MPa]	Specific strength [kN·m/kg]	Elastic modulus [GPa]	Specific stiffness [10^6^ m^2^ s^−2^]
*BC-mycelium_70°C*	1208.38 ± 29.82	1.72 ± 0.59	1.42 ± 0.50	1.10 ± 0.17	0.91 ± 0.23
*mycelium_70°C* (control)	980.67 ± 84.77	1.14 ± 0.13	1.16 ± 0.07	0.59 ± 0.15	0.61 ± 0.17
*BC-a*	2256.41 ± 102.56	9.71 ± 0.05	4.31 ± 0.17	0.05 ± 0.01	0.02 ± 0.00
*BC-b*	2393.16 ± 210.75	35.89 ± 4.77	15.13 ± 2.62	0.34 ± 0.04	0.15 ± 0.03
*BC-c*	2857.41 ± 198.57	76.43 ± 29.58	27.55 ± 12.66	11.91 ± 3.29	4.20 ± 1.20
*BC-d*	1060.85 ± 171.98	30.27 ± 9.64	28.73 ± 8.87	1.25 ± 0.69	1.14 ± 0.63
*BC-e*	1957.67 ± 145.67	14.67 ± 0.36	7.55 ± 0.72	0.84 ± 0.35	0.44 ± 0.20

The standard deviation was based on measurements of quintuple specimens (mean ± one standard deviation)

Wet BC sheets (*BC-a*), in the state directly after harvesting, felt very strong, sturdy and it was impossible to tear them apart by hand-pulling force (Fig. 2a). At the same time, they felt flexible, and folding was possible without issues. During testing, these samples were difficult to clamp and as a result, the water pressure, caused by the hydraulic clamps, resulted in some of the individual samples breaking close to the clamp, while others experienced a gradual break over their length, combined with the sample's narrowing (Poisson). Nevertheless, these samples were quantified to have an average ultimate strength of 9.71 MPa (Table 2). For air-dried samples (*BC-b*), a more brittle and less sturdy material was observed as compared to *BC-a*. These samples were relatively thin, presumably because the three-dimensional structure of the BC network had collapsed. In addition, while drying, the material shrank, resulting in uneven widths and the formation of wrinkles. Yet, *BC-b* samples still maintained a flexible appearance, and the material did not break upon folding and as compared to undried samples, they were characterized by a significantly higher tensile strength (Table 2; Fig. 4a). Next, two sample types were prepared by drying while heat-pressing, either directly upon harvesting (*BC-c*) or by performing this with BC sheets that were already air-dried (*BC-d*). The texture of both heat-pressed samples (*BC-c* and *BC-d*) was very smooth, and the sheets felt homogeneous and robust, although brittle. A considerable increase in tensile strength was observed for heat-pressed samples (*BC-c*) (Table 2; Fig. 4a), presumably due to the cross-linking effect of the heat treatment. On the other hand, heat-pressing samples that were already dry (*BC-d*) did not significantly affect tensile properties. Finally, mechanically disintegrated BC sheets were made (*BC-e*), containing small holes, which were unevenly distributed and causing failures during mechanical tests. Similar as for heat-pressing of dried BC (*BC-d*), blending of air-dried samples (*BC-e*) did not improve ultimate stress but instead enhanced stiffness compared with *BC-b* (Table 2; Fig. 4b). Overall, pure BC sheets were characterized by a much higher tensile strength than mycelium composite materials (Table 2; Fig. 4a).

**Fig. 5:**
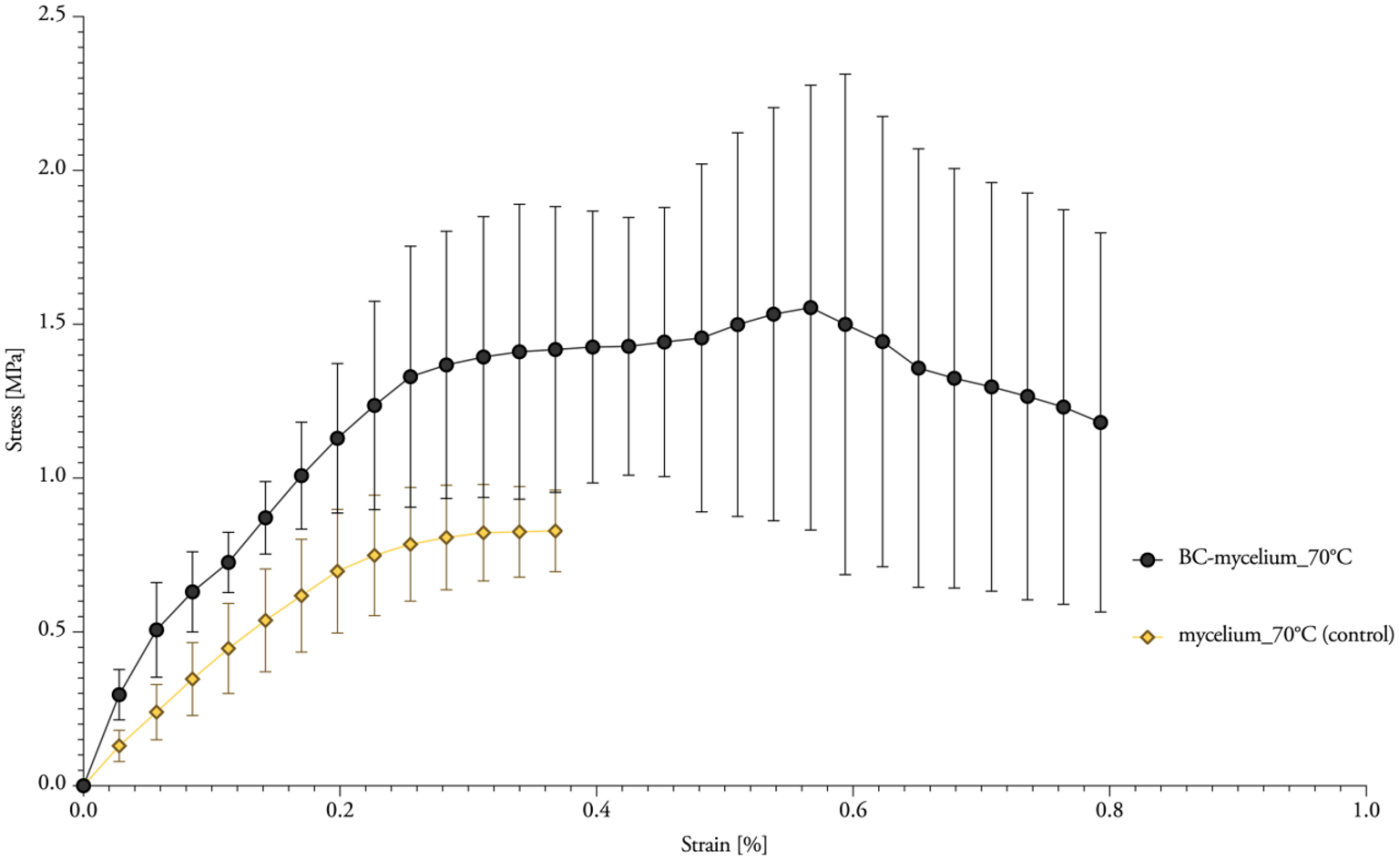
Stress–strain curves from tensile tests of BC-mycelium composites. The standard deviation is performed with quintuple specimens (mean ± one standard deviation)

The addition of BC into mycelium materials resulted in an increased tensile strength of 1.72 MPa (Tσ = 1.42 MPa) and elastic modulus of 1.10 GPa (T_E_ = 0.91 10^6^ m^2^ s^−2^) (Table 2; Figs. 5 and 4a, b). Yet, the values of *BC-mycelium_70°C* are not significant different from *mycelium_70°C*.

### Tensile properties of BC-mycelium composites perpendicular to the surface

**Fig. 6:**
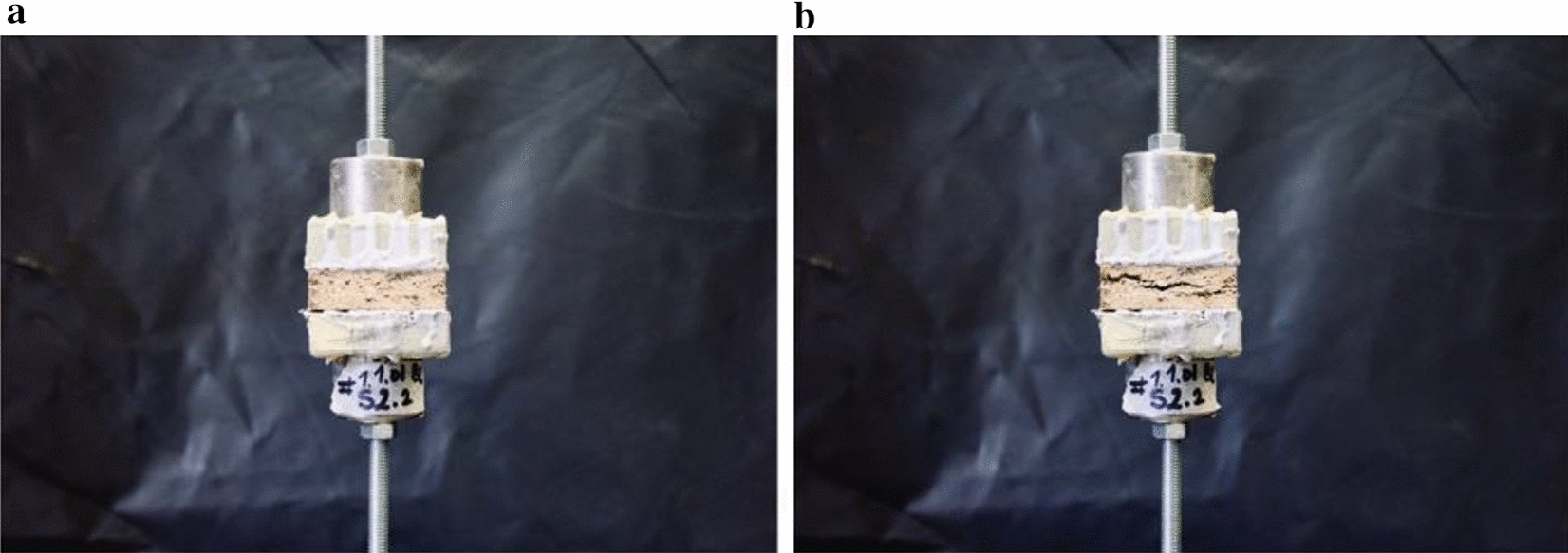
Set-up of the mechanical test for internal bond analysis (tensile behaviour perpendicular to the surface). **a** Sample *BC-mycelium_70°C* before tension. **b** After tension

**Fig. 7:**
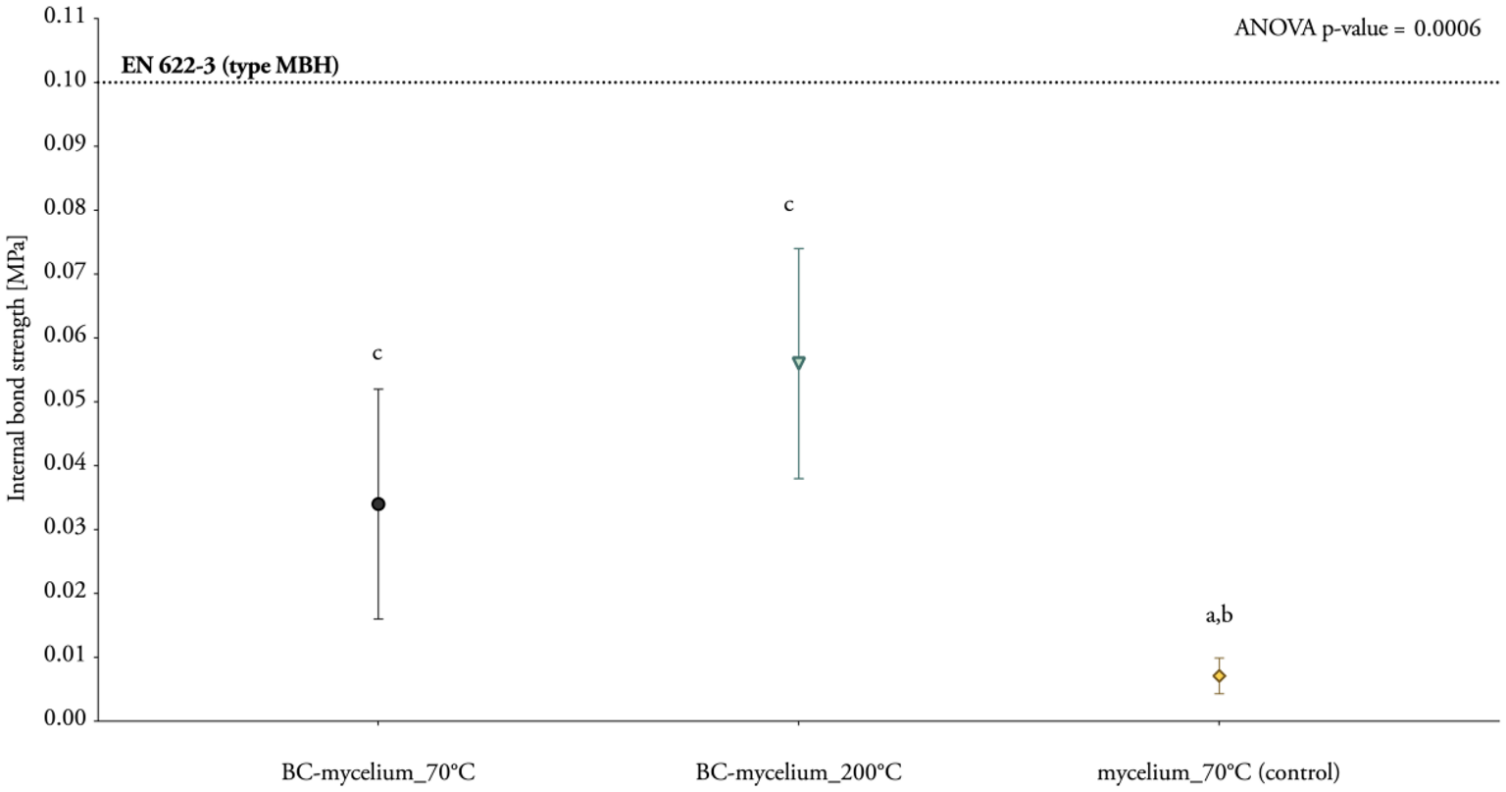
Tensile properties perpendicular to the surface, representing the internal bond strength of BC-mycelium materials. Labels with different letters indicate a statistical difference (p < 0.05) among the specimens

Another key determinant of the mechanical strength of composite materials is the internal bond strength, which is in the case of BC-mycelium composites determined by the adhesion of the BC and mycelium to each other and to the hemp fibres. The internal bond strength of the BC-mycelium particle board samples was measured with a mechanical test quantifying tensile strength perpendicular to the surface (Table 3; Figs. 6, 7). As compared to mycelium composite specimens lacking BC, the internal bond strength of BC-mycelium composites was significantly higher (Table 3; Fig. 7). The highest internal bond strength was achieved for the samples heat-pressed at 200 °C (*BC-mycelium_200°C*), with the ultimate tensile strength being eight times higher as compared to the control samples. To a smaller extent but also significant, *BC-mycelium_70°C* samples performed fivefold better than the control samples. Altogether, these results demonstrate that the addition of BC to the composites positively affects internal bond strength (Table 3; Fig. 7). However, despite this increased performance, the BC-mycelium materials still do not meet medium boards’ requirements as defined in EN 622–3 (2004) (type MBH) (Fig. 7).

**Table 3:** Overview of the internal bond strength and stiffness of BC-mycelium materials

Label	Dry density [kg/m^3^]	Ultimate strength [MPa]	Specific strength [kN·m/kg]	Young’s modulus [GPa]	Specific modulus [10^6^ m^2^ s^−2^]
*BC-mycelium_70°C*	531.10 ± 39.17	0.034 ± 0.02	0.07 ± 0.04	0.005 ± 0.002	0.010 ± 0.003
*BC-mycelium_200°C*	456.82 ± 13.26	0.056 ± 0.02	0.13 ± 0.04	0.007 ± 0.002	0.014 ± 0.006
*mycelium_70°C* (control)	492.32 ± 45.40	0.007 ± 0.003	0.01 ± 0.006	0.005 ± 0.002	0.01 ± 0.0007

The standard deviation was based on measurements of sextuple specimens (mean ± one standard deviation)

## Discussion

The mechanical characteristics of mycelium composites were shown to be slightly better, specifically the internal bond, upon adding BC. However, the fabrication method by heat-pressing at 200 °C improved the mechanical properties significantly, also with respect to other published data of mycelium materials without additives. For example, the flexural strength and modulus of heat-pressed rapeseed and cotton mycelium materials ranges between 0.62 and 0.87 MPa and between 0.03 and 0.07 GPa, respectively [6], values that are much lower as compared to those determined for BC-mycelium composites in this study (Table 1 and Fig. 3). The addition of BC to mycelium composites provided slightly better mechanical properties than the use of cellulose nanofibrils extracted from plant material [13]. Indeed, Sun et al*.* reported, for a similar composition (90% wood particles, 7.5% mycelium and 2.5% cellulose nanofibrils), a flexural strength of 2.73 MPa and a modulus of elasticity at 0.33 GPa [13] as compared to 2.94 MPa and 1.10 GPa, respectively, in this study (Tables 1 and 2). However, direct comparison is challenging since the materials were produced and processed differently.

In the procedure that we employed to fabricate the particle boards, heat pressing is a crucial step. It enhances the material properties, not only for the BC-mycelium composites, but also for mycelium composites without BC. A heat-press treatment of BC-mycelium composites at 200 °C resulted in better mechanical characteristics than at 70 °C (Tables 1, Table 3; Figs. 3, 7). Indeed, heat pressing was previously reported to be a major factor in increasing tensile strength and modulus of mycelium-based materials, relative to cold-pressing or non-pressing [6]. As another example, non-pressed cotton materials were reported to have flexural strengths in the range of 0.007–0.026 MPa [42] and non-pressed mycelium materials made from crop residues and coated with edible films (carrageenan, chitosan and xanthan gum) were reported to have a flexural strength of 0.01 MPa [43]. These low flexural strengths can be explained by a higher porosity and lower density in non-pressed materials. Heat-pressing not only leads to a densification of the material, but also to the polymerisation of lignin [44] present in the hemp fibres. Fibres that are first oriented randomly are now pressed more horizontally into the plane of the panel. Furthermore, the heat causes the formation, but also breaking and reformation, of hydrogen bonds between amino acids in the mycelium and fibres in addition to esterification, leading to a densely packed substrate [44, 45]. It can be expected that cellulose nanofibrils positively contribute to this phenomenon and that additional hydrogen bonds are formed, thereby leading to an enhanced strength and toughness of BC-mycelium materials.

It was previously reported that mycelium composites are typically characterized by a low internal bond strength. For example, in the study of Sun et al. the internal bond strength of mycelium composites without nanocellulose additives was too weak to be measured in a test [13]. In contrast, another study reported an internal bond strength between 0.05 and 0.18 MPa for mycelium-cotton stalk composites fabricated by heat pressing [46]. In this work, we show that the addition of BC improves the internal bond strength, albeit not meeting the required strength according to the standard (Fig. 7). This result is comparable to that of mycelium composites with plant-derived cellulose nanofibrils, for which an internal bond strength of between 0.03 and 0.06 MPa was achieved [13].

Depending on the research, in literature, the strength of pure BC reaches values between 50 and 100 MPa [28], or remains below 50 MPa [47], while this work showed a tensile strength of BC between 9.7–76.4 MPA. The tensile strength of sample *BC-c* (heat-pressed) even reaches a strength close to Nylon (average 79.4 MPa) [48]. Sample *BC-b* has a tensile strength similar to High Density Polyethylene (HDPE) Film Grade (average 36.9 MPa) [49].

The strength and stiffness results of mycelium materials presented in this work should be considered an indication of the impact of specific parameters such as the addition of BC fibrils and heat-pressing. The aim of this work was to comb the spectrum of possibilities and propose valuable directions for further research.

## Conclusions

The findings presented in this study contribute to existing data on the mechanical properties of mycelium materials. In line with the initial research hypothesis, it is shown that the addition of organic BC fibrils to mycelium materials results in enhanced mechanical properties, more specifically the internal bonding improved significantly. Overall, this work suggests that the fabrication method of the particle boards by heat pressing at high temperature impacts the mechanical properties more significantly than the BC additive. The addition of BC contributes to the composites' overall heterogeneity and leads to mycelium composites with more tuneable mechanical properties. Regulating their mechanical properties remains a challenge, especially since their consistency depends on the biological variability of the organism and the biowaste feedstock. Combined, the mycelium, bacterial cellulose, fibres, and type of drying treatment all contribute to the overall mechanical anisotropy of the composites. The findings presented in this study for BC-mycelium hybrids extend existing data about the mechanical properties of mycelium materials obtained in previous studies.

## Methods

### Microbial strains used in this work

The fungal strain *T. versicolor* M9912 was purchased from Mycelia bvba (Nevele, Belgium) under the form of mycelium spawn. It was conserved on a grain mixture at 4 °C in a breathing Microsac bag (Sac O2 nv, Nevele, Belgium).

A *K. xylinus* bacterial strain was isolated from a commercial SCOBY (symbiotic culture of bacteria and yeast) (Fig. 1a) by enriching it in a Hestrin and Schramm (HS) culture medium designed specifically for cellulose-producing bacteria [50] (Fig. 1b), which contained 20 g/L glucose, 5 g/L peptones, 5 g/L yeast extract, 2.7 g/L Na_2_HPO_4_ and 1.15 g/L citric acid. Bacterial species identification was performed by 16S rDNA amplification and Sanger sequencing.

### Fabrication of bacterial cellulose sheets

A starter culture of BC-producing *K. xylinus* was obtained by cultivating the strain in 250 mL HS medium in an Erlenmeyer flask during 10 days at 30 °C in the dark. A white gelatinous substance of intertwined cellulose fibrils accumulated at the surface of the liquid, and in the rest of the liquid, a cloudy loose structure of cellulose appeared (Fig. 1c). After the incubation, a BC pellicle of 5 mm was obtained, and the culture was stored at 4 °C. Next, to prepare BC sheets with a larger surface, Pyrex® glass dishes with dimensions of 40 × 27 cm were covered with aluminium foil and sealed with tape before being sterilised in an autoclave at 121 °C for 15 min. After cooling down, the aluminium foil was carefully cut open in one corner in a laminar flow unit, and 250 mL HS medium was poured in slowly. This was inoculated with 25 mL of the starter culture (Fig. 1d), the aluminium foil was closed again, sealed with tape and the dish was incubated at 30 °C for 10 days in the dark. The bacteria developed BC sheets at the liquid–air interface and once a thickness of about 10 mm was reached, the obtained BC sheets were harvested and rinsed with deionised water before further treatment (Fig. 1e).

Five different types of pure BC samples were prepared: i) a plain undried sheet (sample BC-a) was set aside and immersed in ethanol during storage to avoid contamination (morphological changes may have occurred with the solvent exchange); ii) a sheet was air-dried on a wooden plank between layers of absorbing tissues (sample BC-b); iii) a wet sheet was directly heat-pressed at 190 °C during 20 min (sample BC-c); iv) a sheet was first air-dried followed by heat-pressing at 170 °C during 3 min (sample BC-d) and v) the equivalent of the four prior samples was mixed using a lab blender, spread out in a rectangle shape and left to air-dry during several days while regularly flipping over (sample BC-e).

### Fabrication of hybrid BC-mycelium composite material samples

The BC-hemp substrate was prepared by placing 5–25 mm hemp hurds (Aniserco S.A, Groot-Bijgaarden, Belgium) in heat-resistant bags (Fig. 1g) and autoclaving these at 121 °C for 20 min (Fig. 1h). The bags were then left to cool down for 24 h. The BC sheet was rinsed several times to avoid bringing over acidic residues in the substrate. Then, a BC sheet of 30 g was mechanically disintegrated by mixing it with a lab blender during 5 min after adding 250 mL fresh HS medium and 350 mL deionised sterile water (Fig. 1f, b) before mixing it with 200 g hemp fibres (Fig. 1i). This mixture was incubated for 5 days at 30 °C on a rotary shaker rotating at a speed of 105 rpm (Fig. 1j).

To initiate the formation of mycelium-based material, the fibre mixture was supplemented with 10 wt% of *T. versicolor* mycelium spawn (Fig. 1k) and placed in bags with a depth-filtration system that allowed for air exchange. During a first growth phase, the bags were incubated at 26 °C with a relative humidity of 60%. Every day, the bags were kneaded manually to stimulate the strengthening of the mycelium (Fig. 1l). After 5 days of mycelial growth, the substrate was crumbled by hand and transferred to Microbox containers (SacO2, Deinze, Belgium) with a depth-filtration system on top (185 × 185 × 78 mm). These containers were further incubated at 26 °C during 10 days. Subsequently, the samples were removed from the containers that served as moulds and were then incubated again for 2 days to achieve homogeneous colonisation on the sides that had been in contact with the container.

### Particle board fabrication

The BC-mycelium samples were compressed with an Instron 5900R with an oven built around (Fig. 2c), by applying a maximum force of 30 kN at 2 kN/min. When a displacement of 50 mm was reached, the load was maintained for 10 h while incubating at 70 °C (first batch) or 200 °C (second batch). The obtained particle boards were then stored at 21 °C and 65% relative humidity (RH) during 3 to 4 weeks before testing. Finally, the samples were cut with a thin blade saw into smaller specimens at dimensions required for the mechanical tests (170 × 50 mm for static bending tests, 50 × 50 mm for tensile strength tests perpendicular to the surface and 180 × 30 mm for tensile strength tests parallel to the surface) (Fig. 2d).

### Bending behaviour analysis

Since no standard exists for testing mycelium materials, bending behaviour tests were performed according to specifications of norms that were expected to result in similar properties. Given that the characteristics of mycelium materials are typically similar to those of foam and wood-based panels, the bending behaviour of the BC-mycelium composite specimens was determined according to the following standards: ISO 16978—*Wood-based panels—Determination of modulus of elasticity in bending and of bending strength* [51] and ISO 12344—*Thermal insulating products for building applications – Determination of bending behaviour* [52]. Three-point static flexural tests were performed on test specimens of 170 × 50 mm using an Instron 5900R load bench with a load cell of 10 kN. A loading speed of 2.5 mm/min was applied. These tests were performed in triplicate.

The bending strength ƒ_m_, of each test piece, was calculated from the formula [51]:

1 $${\text{f}}_{m} = \frac{{3F_{max} l_{1} }}{{2bt^{2} }}{\text{ [MPa],}}$$

with F_max_ is the maximum load [MPa], l_1_ the distance between the centres of the supports [mm], b the width of the test piece [mm] and t the thickness of the test piece [mm]. The modulus of elasticity E_m_, is calculated from the formula [51]:

2 $$E_{m} = \frac{{l_{1}^{3} \left( {F_{2} - F_{1} } \right)}}{{4{ }bt^{3} \left( {a_{2} - a_{1} } \right)}}\;[{\text{MPa}}],$$

where l_1_, b and t are the dimensions as defined above, F_2_-F_1_ is the linear portion of the load–deflection curve [N], F_1_ is 10% and F_2_ is 40% of the maximum load. The term a_2_—a_1_ represents the increment of deflection at the mid-length of the test piece (corresponding to F_2_ – F_1_). Thickness, length and width was measured with a digital calliper for all samples.

### Analysis of tensile behaviour parallel to the surface

**Fig. 8:**
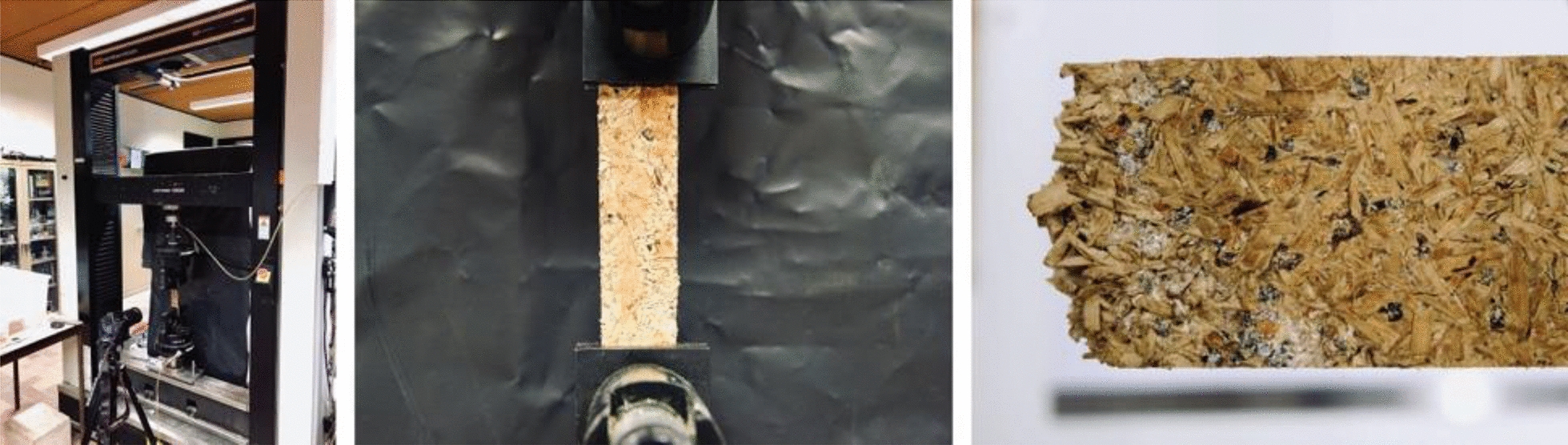
Test set-up (left), BC-mycelium composite (middle), particle board after tension testing (right)

Tensile strength parallel to the surface was measured according to ASTM 1037 – *Standard Test Methods for Evaluating Properties of Wood-Base Fiber and Particle Panel Materials* [53]*.* In this case, measurements were performed for five replicate specimens with dimensions of 170 × 30 mm, again using an Instron 5900R load bench with a load cell with a maximal capacity of 10 kN but with a loading speed of 1 mm/min (Fig. 8). The load–displacement curve was converted to a stress–strain curve, using the following formulas to calculate the stress σ and the strain ε [53]:

3 $$\sigma = \frac{F}{A}\;[{\text{MPa}}]$$

and

4 $$\varepsilon = \frac{{{\Delta }L}}{Lo}\;[ - ]$$

where σ is the stress [N/mm^2^], F is the applied force [N], A is the original cross-section of the specimen [mm^2^], ΔL is the elongation of the specimen of the loading surfaces [mm] and L_o_ is the original length of the test piece [mm]. The ultimate tensile strength and elastic modulus were calculated using the following formulas [53]:

5 $${\upsigma }_{u} { } = { }\frac{{F_{max} }}{A}\;[{\text{MPa}}],$$

and

6 $$E = \frac{\sigma }{\varepsilon }\;[{\text{GPa}}],$$

where ${\upsigma }_{u}$ is the ultimate tensile strength [MPa], F is the maximum load [N], A is the original cross-section of the specimen [mm^2^], E is the Young’s modulus and is measured in the linear portion of the load–deflection curve, [GPa], σ is the uniaxial stress and ε is the strain. Finally, the specific strength and modulus were calculated using the following formulas:

7 $$T_{\sigma } = \frac{{{\upsigma }_{{\text{u}}} }}{{\uprho }}\;\left[ {{\text{kN}}\cdot{\text{m}}/{\text{kg}}} \right],$$

and

8 $$T_{E} = \frac{E}{\rho }\;\left[ {{1}0^{{6}} {\text{m}}^{{2}} {\text{s}}^{{ - {2}}} } \right],$$

where $T_{\sigma }$ is the specific tensile strength [kN·m/kg or or MPa/(g/cm^3^)], ${\upsigma }_{u}$ is the ultimate tensile strength [MPa], $\rho$ is the density [g/cm^3^], $T_{E}$ is the specific Young’s modulus [10^6^ m^2^ s^−2^ or GPa/(g/cm^3^)] and E is the Young’s modulus [GPa].

### Dry density and moisture content

The density was calculated following ISO 9427—Wood-based panels—Determination of density by taking the ratio of the oven-dry mass over the volume. The moisture content was calculated with the formula [54]:

9 $$M = \frac{{\left( {W_{w} - W_{d} } \right){*}100}}{{W_{w} }}\;[\% ]$$

where M is moisture content [%], w_w_ is wet weight [g], w_d_ is oven-dry weight [g].

### Analysis of tensile behaviour perpendicular to the surface

A test was performed to determine tensile behaviour perpendicular to the surface thereby assessing the cohesion (internal bond) of the material (Fig. 6). Since the particle boards were produced by compressing the material in one direction, the tensile behaviour is expected to be different for parallel compared with the perpendicular forces. This test was executed according to *EN 319:1993 Particle boards And Fibreboards. Determination Of Tensile Strength Perpendicular To The Plane Of The Board* [55]. Specimens with dimensions of 50 × 50 mm were glued on aluminium loading blocks and after 24 h curing, the block was mounted into the grips of an Instron 5900R load bench with a maximal capacity of 10 kN. The specimens were loaded at a uniform motion rate of 0.5 mm/min until failure occurred. These tests were performed in duplicate.

Based on these measurements, tensile strength was calculated by:

10 $${\upsigma }_{IB} { } = { }\frac{F}{A}\;\left[ {{\text{MPa}}} \right]$$

where ${\upsigma }_{IB}$ is the tensile strength perpendicular to the surface (internal bond strength), F is the maximum load and A is the area (length x width) of the specimen.

### Statistical analysis

The statistical evaluation was performed in Microsoft Excel and graphed with GraphPad Prism (version 8.1.2). Data were checked for normality (p ≥ 0.05) using a Kolmogorov–Smirnov test. A one-way analysis of variance (ANOVA) was used for normal data, and significant differences were considered at p ≤ 0.05. The multiple comparisons test for normal data was generated based on Tukey’s family error rate. For non-parametric data, the Kruskal–Wallis test was conducted, and significant differences were considered at p ≤ 0.05. The Dunn’s multiple comparison test was used for the non-parametric data. Tree-point bending tests had triplicate specimens, tensile tests had quintuple specimens, and internal bond tests had sextuple specimens.

## Data Availability

The datasets used and/or analysed during the current study are available from the corresponding author on request.
